# Reconciling Color Vision Models With Midget Ganglion Cell Receptive Fields

**DOI:** 10.3389/fnins.2019.00865

**Published:** 2019-08-16

**Authors:** Sara S. Patterson, Maureen Neitz, Jay Neitz

**Affiliations:** ^1^Department of Ophthalmology, University of Washington, Seattle, WA, United States; ^2^Neuroscience Graduate Program, University of Washington, Seattle, WA, United States

**Keywords:** primate retina, color vision, color perception, computational vision, linking hypotheses, cone photoreceptor, retinal ganglion cells

## Abstract

Midget retinal ganglion cells (RGCs) make up the majority of foveal RGCs in the primate retina. The receptive fields of midget RGCs exhibit both spectral and spatial opponency and are implicated in both color and achromatic form vision, yet the exact mechanisms linking their responses to visual perception remain unclear. Efforts to develop color vision models that accurately predict all the features of human color and form vision based on midget RGCs provide a case study connecting experimental and theoretical neuroscience, drawing on diverse research areas such as anatomy, physiology, psychophysics, and computer vision. Recent technological advances have allowed researchers to test some predictions of color vision models in new and precise ways, producing results that challenge traditional views. Here, we review the progress in developing models of color-coding receptive fields that are consistent with human psychophysics, the biology of the primate visual system and the response properties of midget RGCs.

## Introduction

**FIGURE 1:**
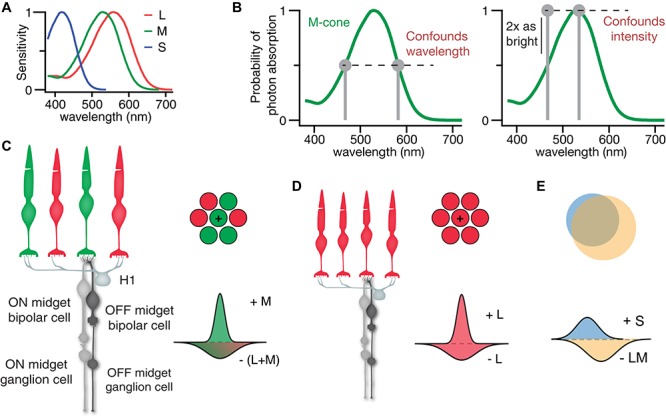
Color-coding receptive fields of the primate retina. **(A)** The normalized spectral sensitivities of human L-, M-, and S-cone photoreceptors. **(B)** Demonstration of monochromatic lights that elicit the same probability of photon absorption, and thus elicit the same response from M-cones. On left, 467 and 582 nm lights are indistinguishable, despite a 115 nm difference. On the right, a 467 and 535 nm lights of different intensities can be confounded by adjusting the intensity of the 467 nm light. **(C)** Circuit diagram of the upstream input to ON and OFF midget RGCs. **(D)** Same as in **(C)**, but for the midget RGCs of a dichromat. **(E)** Small bistratified RGC receptive field diagram illustrating the lack of perfectly coincident spectrally opponent receptive fields as required for pure color cells.

The first stage of visual processing occurs in the retina, an outpost of the brain located at the back of the eye. Under photopic conditions, photons of light are absorbed by three types of cone photoreceptor (Figure 1A), processed by five main classes of retinal neuron, then visual signals are conveyed to the brain by the axons of retinal ganglion cells (RGCs; Wässle, 2004). Midget RGCs make up a large majority of all RGCs in the central retina, where each L- and M-cone provides the sole direct input to an ON and OFF midget RGC circuit (Figure 1C; Wässle et al., 1990, 1998; Kolb and Marshak, 2003).

The midget RGC receptive field has a center-surround organization (Kuffler, 1953). In the central retina, this receptive field compares the photon catch in the single L- or M-cone center to the photon catch in neighboring L/M-cones in the surround (Figure 1C). Since this configuration compares the activity of cones that differ in both spatial location and spectral sensitivity, midget RGCs have been implicated in both color and spatial vision (Schiller et al., 1990; Martin et al., 2011). Mammalian RGCs have been described as acting as feature detectors, with different types showing specificity for motion, form or color conferred by the spatial, spectral, and temporal characteristics of their receptive field (Field and Chichilnisky, 2007; Gollisch and Meister, 2010; Baden et al., 2016). Here, we review evidence for the role of midget RGC receptive fields as the first step for detection of two elementary visual features, (1) hue detectors which encode information about spectral reflectances of surfaces as red, green, blue and yellow percepts, (2) high acuity edge detectors which encode the boundaries of objects as required for form vision.

Because their receptive fields exhibit both spectral and spatial opponency, midget RGCs respond to both chromatic and achromatic edges and thus confound the two (Wiesel and Hubel, 1966). Like all RGCs, midget RGCs encode and transmit information to the brain in binary, as all-or-nothing action potentials. A downstream neuron has no way of knowing, from an individual midget RGC’s response, whether the midget RGC responses represent the chromatic or spatial structure of a stimulus. At the level of perception, however, we can distinguish between achromatic and equiluminant chromatic edges, even though individual midget RGCs cannot. How and where the spectral and spatial information encoded by midget RGCs is extracted remains one of the most important unanswered questions of primate vision.

Midget RGCs provide, arguably, the best model for linking low-level receptive fields to perception. Understanding how color and spatial information are encoded may provide insight into general organizational principles employed by neural circuits to parse specific features of a stimulus. Furthermore, restoration of color and spatial vision are an important goal for retinal prosthetics, some of which must replace the upstream circuitry that defines the midget RGC receptive field (Yue et al., 2016). Efforts to restore these fundamental aspects of visual perception may benefit from a better understanding of how they are computed in normal vision.

## Receptive Fields

All receptive fields are built from the photoreceptor outputs (Figure 1A). The photoreceptors’ output encodes a single variable: the number of photons absorbed (Rushton, 1972; Baylor et al., 1987). An important implication is that wavelength and intensity are interchangeable and, under the right conditions, any two lights differing in wavelength can be “substituted silently” for each other (Estevez and Spekreijse, 1982). For example, the probability of photon absorption by an M-cone is the same for 467 and 582 nm lights, thus the response of the M-cone shown in Figure 1B to the two lights will be indistinguishable. Meanwhile, a 535 nm light with twice the probability of photon absorption can be matched by doubling the intensity of the 467 nm light.

The visual system extracts information about wavelength and spatial contrast by virtue of receptive fields that compare the outputs of multiple cones. The basic computation for extracting wavelength is a comparison between cones of different spectral types, while spatial contrast requires comparing neighboring cones at different spatial locations, regardless of type (Calkins and Sterling, 1999). The characteristics of receptive fields form the foundation of each color vision model discussed here.

## What Is the Optimal Receptive Field for Spatial Vision?

Because midget RGCs are implicated in high acuity form vision, any discussion of their color-coding role must also include their role in spatial-coding. The first step of spatial vision requires delineating the boundaries of objects, essentially performing an edge detection task.

### Spatial Opponency

By comparing the relative activity of cones at different locations, spatially opponent receptive fields signal spatial contrast rather than raw quantal catch (Srinivasan et al., 1982). For low-level edge detectors, circularly symmetric center and surround receptive fields are optimal and will provide sensitivity to all edges, regardless of their orientation (Marr and Hildreth, 1980).

### Spectral Opponency

While spatial vision is sometimes assumed to operate only on light intensity (Marr, 1982; Billock et al., 1996), equiluminant edges are also common in natural scenes (Hansen and Gegenfurtner, 2009). Accordingly, an optimal edge detector would be sensitive to all edges regardless of whether the edge is defined by a change in wavelength or intensity. Thus, an optimal edge-detecting receptive field might not just be spatially opponent, but also spectrally opponent. In this case, the purpose of spectral opponency is not to signal the hue of a surface but rather an edge defined by spectral contrast.

## What Is the Optimal Color-Coding Receptive Field for Hue Perception?

In the natural world, most colors we perceive are from lights reflected from objects. The purpose of hue perception is to provide information about the surface reflectance of objects, which, in turn, tells us about their internal contents or state. For example, we know the ripeness of fruit and when children are getting sunburned from their surface colors. However, there are significant challenges to this task. Individual cones themselves are not selective for the *distribution* of wavelengths reflected from a surface. If L-cones are active, light could be coming from a red surface reflecting only long wavelengths, a yellow surface reflecting both middle and long wavelengths, a violet surface reflecting both short and long wavelengths or a white surface reflecting all wavelengths. In addition, information from any individual cone will be further confounded by the spectral characteristics of the illuminant. For example, the amount of illumination from blue sky light relative to direct sunlight changes throughout the day. As a result, the illuminant color can vary from blue to yellow (Foster, 2011; Pauers et al., 2012; Spitschan et al., 2016; Woelders et al., 2018). The ideal receptive fields for serving hue perception would be designed to help extract surface spectral reflectance independent of the illuminant. Here we discuss the features of theoretical receptive fields optimized to overcome the challenges associated with consistently signaling hue, independent of any underlying neural substrates.

### Spectrally Opponent

Color vision is the ability to discriminate between different wavelengths, independent of intensity (Jacobs, 2018). Receptive fields with spectrally opponent interactions can extract wavelength information and thus *carry color information* (Paulus and Kroger-Paulus, 1983; Neitz and Neitz, 2011; Chang et al., 2013). However, cone opponent receptive fields are not necessarily optimized for hue perception.

### Spatially Coextensive

**FIGURE 2:**
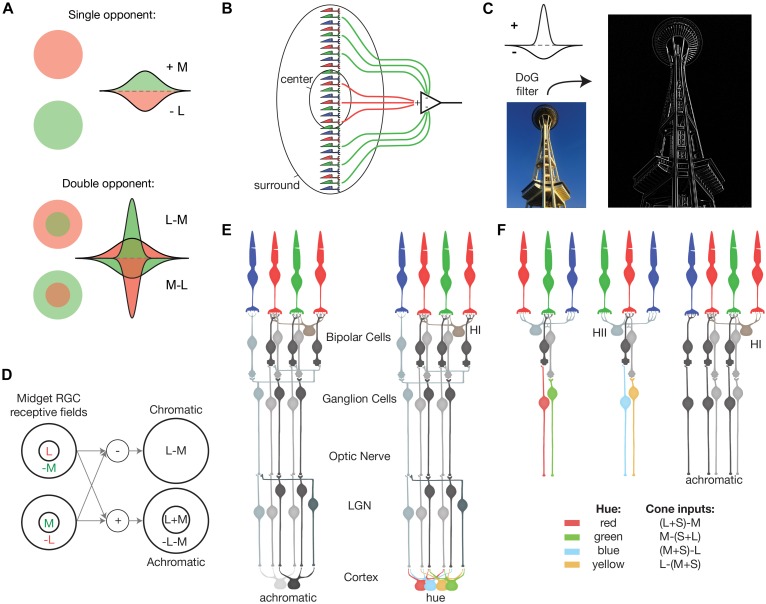
Models of receptive fields encoding form and color vision. **(A)** Diagrams of single and double opponent receptive fields. Adapted from Daw (1973). **(B)** Putative receptive field formed by selective wiring. Adapted from Wiesel and Hubel (1966). **(C)** Edge detection performed by convolution of an image with an achromatic center-surround receptive field, or Difference of Gaussians filter. **(D)** Circuit for “de-multiplexing” chromatic and achromatic information from midget RGC receptive fields. Adapted from Derrico and Buchsbaum (1991). **(E)** Circuit proposed by the multi-stage color model (De Valois and De Valois, 1993). **(F)** Circuit proposed by the parallel processing model (Neitz and Neitz, 2016).

The first receptive field proposed to create a “pure color cell,” was the single opponent receptive field, which exhibits spectral opponency without any spatial opponency (Figure 2A). Also called spatially co-extensive or Type II (Wiesel and Hubel, 1966; Crook et al., 2009), this receptive field provides *color selectivity*, the ability to extract spectral information unconfounded by spatial information. Spatially co-extensive, spectrally opponent receptive fields like Figure 2A would be theoretically color selective in that they respond to chromatic stimuli, but not achromatic patterns. However, these receptive fields act as simple wavelength detectors and cannot compensate for the changes in illuminant discussed above.

### Double Opponency

To consistently signal hue, an optimal color-coding receptive field must compensate for the changes in illuminant discussed above. Double-opponent receptive fields, superimposing two opposing, spectrally and spatially opponent receptive fields (Figure 2A) have been proposed to help provide this *color constancy* (Daw, 1973; D’Zmura and Lennie, 1986). Double opponent receptive fields exploit the fact that, in the natural world, hue typically changes abruptly at object boundaries while illumination changes slowly across a visual scene. When the center receives light from the edge of an object surface, some light falling in the surround is reflected from other objects in the scene under the same illuminant. If the illuminant changes to have more long-wavelength light, the increased L-cone stimulation in the center is opposed by greater L-cone stimulation in the surround, and ideally, the change in illumination is removed from the visual signal. Thus, double opponent receptive fields confer sensitivity to chromatic contrast at the edges of objects while remaining relatively insensitive to global changes in illumination.

### Trichromatic

Normal humans are trichromats and a special requirement of optimal color coding for trichromats is that the receptive fields must compare all three cone types. This is because for neurons comparing only two out of the three cone types, a change in activity in the unsampled cone will not change the hue signaled by that neuron. For example, an L vs. M opponent neuron without S-cone input, as in Figure 2A, cannot discriminate between a red surface reflecting only long wavelengths and a violet surface reflecting both long and short wavelengths (Fuld et al., 1981).

### Low Spatial Resolution

If the ideal retina is composed of multiple types of feature detectors, spatial constraints must be considered, and the relative density of any one type should be no higher than required to serve its specific function. The color of a surface tends to be consistent all across it. Thus, in contrast to spatial vision, that requires a high density of detectors to capture the fine details of the shape of objects, hue detectors can accurately capture surface colors using a much lower resolution array of detectors. In summary, the ideal trichromatic hue-encoding system is a relatively sparse array of receptive fields with structures that are double-opponent and receive input from all three types of cones.

## Interpreting Midget Rgc Receptive Fields

Early models linking L vs. M midget RGCs to visual perception focused on either spatial or spectral opponency in isolation. Models focusing on their spectral opponency emphasized their potential role in encoding red and green hues. In contrast, models accounting only for achromatic spatial opponency lead to the perspective that spectral opponency is an unintended consequence of trichromacy and may be considered “poor engineering” (Marr, 1982).

### Are Midget RGC Receptive Fields Optimal for Hue Perception?

The earliest models followed the first parvocellular LGN (P cell) recordings (De Valois et al., 1966; Wiesel and Hubel, 1966), which have similar receptive field properties as their L vs. M midget RGC inputs. At the time, opponent process theory was still highly controversial (Hurvich and Jameson, 1957) and the discovery of color-opponent neurons in the visual system was groundbreaking. The resulting hypothesis that the parvocellular LGN projections of midget RGCs are responsible for red-green hue perception arguably played a large role in shaping later research. Further, spatial opponency and the resulting responses to achromatic and spatially-structured stimuli were overlooked in many accounts of the physiological basis of hue perception.

In emphasizing, the proposed role of midget RGCs in mediating red-green hue percepts, it was argued that the optimal color-coding receptive field, was one in which an L-cone is surrounded entirely by M-cones, or vice versa. This receptive field, which would seem to require some cone-specific selective wiring, maximizes the spectral difference between the center and surround to maximally decorrelate the outputs of L- and M-cones’ overlapping spectral sensitivities (Figure 1A; Buchsbaum and Gottschalk, 1983; Párraga et al., 2002; Sun et al., 2006). The “selective-wiring” model in Figure 2B was challenged by theoretical studies demonstrating that mixed L/M-cone receptive fields could generate sufficient spectral opponency (Paulus and Kroger-Paulus, 1983; Lennie et al., 1991). Though still debated by some (Lee, 1996; Wool et al., 2018), there is, at most, only a slight functional bias toward selective wiring (Buzás et al., 2006; Field et al., 2010).

A lack of selective wiring may be one argument against the idea that midget RGCs are optimized for hue perception. However, more importantly, from above, the ideal trichromatic hue-encoding system is a relatively sparse array of receptive fields with structures that are double-opponent and receive input from all three types of cones. The common L vs. M midget RGCs do not conform to any of these theoretical features of hue-encoding neurons. While our theoretical discussion cannot rule out a contribution to hue, we can conclude L vs. M midget RGCs, by themselves, are “non-optimal” for hue perception.

### Are Midget RGC Receptive Fields Optimal for Spatial Vision?

Near the fovea, the midget RGC’s receptive field center represents the cone providing direct input to the midget bipolar cell, while the surround is formed by feedback from horizontal cells contacting neighboring cones (Figure 1C; Verweij et al., 2003). This feedback weights each cone’s response by the quantal catch in neighboring cones, essentially subtracting out the mean light level and allowing each individual cone feeding the center of midget RGCs to encode spatial contrast (Jadzinsky and Baccus, 2013). In the central retina, midget RGCs set the limits of human visual acuity (Rossi and Roorda, 2010).

Indeed, theoretical attempts to derive an optimal receptive field for the first step of spatial vision have all converged on the same circularly symmetri center-surround organization (Marr and Hildreth, 1980; Srinivasan et al., 1982; Atick et al., 1992), often modeled as a Difference of Gaussians (Enroth-Cugell and Robson, 1966; Croner and Kaplan, 1995; Dacey et al., 2000). As Figure 2C demonstrates, center-surround receptive fields are ideal edge detectors for encoding spatial contrast.

In contrast to early ideas emphasizing their putative role in color perception, more recent research into the evolution of the primate visual system provides a useful context for a modern understanding of L vs. M midget RGC function. Though sometimes compared to the X-cells of the mammalian retina, there is no true homolog to the midget circuit prior to prosimians (Peng et al., 2019). The midget RGC circuitry evolved before uniform trichromacy (Nathans, 1999). In dichromats, for example, with only S- and L-cones, the midget RGC’s antagonistic center-surround receptive field functions as an achromatic edge detector by comparing the input of a single L-cone to surrounding L-cones (Figure 1D).

### Interim Conclusions

The receptive field structure of L vs. M midget RGCs is consistent with a role in edge detection. Their ability to respond to equiluminant edges defined only by wavelength differences makes visible forms that would be otherwise invisible. Spectral opponency can also increase the signal-to-noise ratio for edges defined by both intensity and wavelength. The idea that spectral opponency in L vs. M midget RGCs could enhance edge detection rather than contribute to color perception raises an important point. A response to wavelength changes does not imply a causal role in hue perception. As introduced above, hue perception requires detectors that will not respond to black-white edges.

In conclusion, while it may be arguable whether or not midget L vs. M RGCs are ideal achromatic encoders, it is indisputable that they are far from ideal for red-green hue encoding. This leaves two major unanswered questions: what is the physiological basis for hue perception and what role do midget RGCs play? Several different theories involving both the spectral and spatial aspects of midget RGC receptive fields have been proposed as tentative answers to this question. We next review the two main classes of explanation: multiplexing and parallel processing.

## Multiplexing Models

The first class of models share the idea that each individual midget RGC does “double duty,” carrying information for both color vision and achromatic spatial vision, which are extracted by circuitry at higher levels of processing in the geniculostriate pathway. It has been said that red-green and black-white percepts are “de-multiplexed” by downstream circuits (Boycott and Wässle, 1999; Lennie and Movshon, 2005). The idea of multiplexing originated as an analog to attempts to efficiently compress chromatic and spatial information for color televisions (Ingling and Martinez-Uriegas, 1983; Derrico and Buchsbaum, 1991).

The most common models, summarized in Figure 2D, combine the outputs of midget RGCs to perform two main transforms: one to extract spectral information by removing spatial correlations and another to extract achromatic spatial information by removing spectral information. The achromatic channels (L + M) sum L- and M-center midget RGC signals to serve as intensity contrast detectors. The putative chromatic channels (L vs. M) difference L-ON-center with M-ON center receptive fields to produce spatially coincident spectrally opponent receptive fields, as discussed above (Figure 2A). Accordingly, achromatic spatial structure will be absent in the chromatic channel, resulting in a low-pass chromatic filter, while the achromatic channel will retain the band-pass spatial tuning necessary for spatial vision.

**FIGURE 3:**
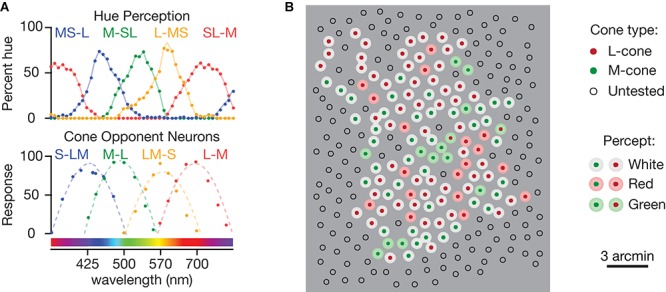
Experiments inspiring the revision of existing color models. **(A)** Spectral sensitivities and the corresponding cone inputs of the mechanisms responsible for red, green, blue, and yellow hues. The data was obtained from a hue scaling experiment, where participants report the percentage of red, green, blue, and yellow. Bottom: Averaged responses of color-opponent LGN neurons, which reflect their color-opponent RGC inputs. Both panels are replotted with wavelength units from De Valois (2004). **(B)** Percepts associated with stimulating individual L- and M-cones in isolation may represent the responses of two types of individual midget RGCs, a larger group of achromatic contrast detectors and a smaller group that function as hue detectors. Adapted from Sabesan et al. (2016).

A separate aspect of one of the best-known versions, the De Valois and De Valois (1993) multi-stage color model, was the need to reconcile the difference in cone inputs measured for L vs. M cone-opponent neurons and the opponent receptive fields required to account for hue perception, illustrated in Figure 3A. The four fundamental hue sensations are often assumed to represent the responses of four groups of hue-encoding neurons. Over the last 50 years, there have been different ideas about the exact nature of the cone inputs to the four fundamental hues. However, a convergence of modern evidence from experiments directly measuring hue perception indicate that all three cone types contribute to each hue in the following combinations: L + S vs. M for red-green and M + S vs. L for blue-yellow, respectively (Figure 3A; Wooten and Werner, 1979; Drum, 1989; Webster et al., 2000a; Schmidt et al., 2016).

One of the great insights of the DeValois and DeValois model was that hue perception requires S-cone inputs to L vs. M opponent pathways (Wooten and Werner, 1979; Drum, 1989; Webster et al., 2000b). As an *ad hoc* solution to the discrepancy between L vs. M midget RGCs and the receptive fields required for hue perception, their multi-stage color model proposed that the necessary S-cone input to an L vs. M channel is accomplished by mixing in the outputs of S-cone opponent neurons (Figure 2E).

### Evaluating the Double Duty Hypothesis

The DeValois and DeValois model was firmly based on the most recent anatomical, psychophysical and physiological results of the time, yet a number of assumptions were necessary where open questions remained. We can now revisit these assumptions in light of the research published in the 25 years since the multi-stage model was first proposed. One example is their explanation of how the required S-cone inputs from small bistratified RGCs are added in the process of building cortical receptive fields for hue perception. More recently, the classification of small bistratified RGCs as single opponent “pure color cells” has been called into question [compare Figures 1E, 2A (Field et al., 2007; Tailby et al., 2010); but see Crook et al. (2009)]. Thus, small bistratified RGCs and their S-ON projections may also confound spatial and spectral information. Moreover, the S-cone ON neurons were later identified as a part of the functionally distinct koniocellular pathway (Martin et al., 1997) and there is no direct evidence for specific circuits combining signals from the koniocellular and parvocellular pathways.

While the theoretical L-M and L + M channels would decorrelate the outputs of midget RGCs, it has been argued that not all decorrelations are created equal (Pitkow and Meister, 2012) and the benefits depend on how these channels are implemented by neural circuitry. In general, however, asking a neuron to perform two jobs simultaneously has been said to ensure that both are done poorly (Sterling and Laughlin, 2017). Moreover, there don’t appear to be any true modern examples of multiplexing RGCs involving two functions performed simultaneously. Perhaps the closest parallel is the fact that the same RGCs serve both photopic and scotopic vision, however, these functions are primarily performed separately under different conditions (Field et al., 2009; Grimes et al., 2014). Other examples of multiplexing RGCs involve one stimulus dimension modulating the encoding of another (Deny et al., 2017), however, this is different from two functions being encoded simultaneously.

The “de-multiplexing” multi-stage models are the result of speculation about the type of computation that would be required to produce selective detectors for wavelength and spatial contrast from combinations of spectrally opponent center-surround neurons, however, they lack firm experimental evidence from cortical physiology (Lee, 2008). They have also been criticized from an image compression standpoint, with the argument that decorrelation of chromatic and spatial information is best done early, ideally before transmission through the optic nerve (Derrico and Buchsbaum, 1991). In contrast, an effort to test de-multiplexing models concluded the two dimensions cannot be disentangled in the early visual system (Kingdom and Mullen, 1995). Moreover, the most successful models based on the “double duty” hypothesis do not make predictions about both spatial and spectral responses (Rider et al., 2018).

The assumption that different aspects of color vision are all based on the same underlying neural substrates (e.g., L vs. M midget RGCs) has resulted in a tendency to expect the visual system to somehow extract hue information from the midget RGCs’ receptive field output. However, the computational complexity required to separate chromatic from spatial information at subsequent stages of visual processing should not be underestimated. One higher stage is proposed to decorrelate spatial and spectral information, a second higher stage to add the required S-cone input (Figure 2E) and yet an additional stage, that has not been incorporated into current de-multiplexing models, to generate the double opponent receptive field structure required to create neurons that are able to contribute to invariant hue-encoding of spectral reflectance.

### Multiplexing in the Light of Information Coding in the Retina

The need to compress RGC axons down to a 2 mm cable is often referred to as an “information bottleneck” within the visual system. Proponents of multiplexing models might claim superiority on this account: combining color and spatial information into one RGC could reduce the number axons in the optic nerve without reducing the transmission of information. Indeed, there are about six to seven million cones in a human eye and only about a million optic nerve fibers (Sterling and Laughlin, 2017). However, this represents the situation in the peripheral retina where convergent input from a large number of cones to each RGC results in a huge reduction in visual acuity relative to what could be supported by the cone mosaic. The loss of spatial information from this convergence is never recovered at higher levels in the visual pathway.

At the time multiplexing models were first proposed, a dominant view on the purpose of retinal function was to reduce redundancy and compress visual information to fit through the optic nerve, with the computations defining visual perception occurring in the cortex (Barlow, 1961). However, contrary to the idea of information compression, in the fovea there is a divergence from cones to RGCs such that the ratio is about 3:1 RGCs:cone. Recent work in non-primate animal models has contributed to a growing appreciation for the diversity of RGC types (Wässle, 2004; Baden et al., 2016) and the sophisticated computations occurring within the retina (Gollisch and Meister, 2010; Wienbar and Schwartz, 2018). Even near the primate fovea, many of the at least 20 different RGC types are represented (Percival et al., 2013; Peng et al., 2019). What failed to be appreciated in the early work on the primate retina is that, with the exception of the midget RGCs, for which there are two for every cone (one ON and one OFF), each of the twenty or more RGC types represents a small percentage of the total. Thus, the retina is a massively parallel processing machine with many different types of RGCs carrying out diverse functions most of which operate at low spatial acuity and require only sparse representations. Thus, as discussed below, it seems plausible that, consistent with the current understanding of the plan of the retina, hue perception could be mediated by a relatively sparse set of RGCs that serve as hue detectors.

Recent considerations of the metabolic cost of information transmission have also questioned the efficiency of compressing information into a smaller set of RGCs, and revealed a more nuanced set of constraints defined not by the number of axons, but by their diameter. RGC axon diameters scale linearly with average firing rate (Perge et al., 2009, 2012). This relationship forms the basis of a law of diminishing returns – metabolic cost increases supralinearly with axon diameter while the information per spike falls as spike rate increases (Rieke et al., 1997; Koch et al., 2006).

A population of parallel neurons, each carrying as much information as possible, is the most efficient coding scheme (Laughlin, 2001). The midget RGC circuit, acting as an edge contrast detector, is already a model of energy-efficient parallel processing – each cone in the central retina contacts a single ON and OFF midget bipolar cell (Figure 1C). This allows baseline activity to remain low while the response ranges of each ON and OFF cell are devoted to signaling increments or decrements, respectively, in parallel (Berry et al., 1997). Theoretically, multiplexing increments and decrements would double the information per axon, thus halving the number of axons while increasing axon diameter (and thus energetic cost) fourfold (Sterling and Laughlin, 2017). Taking these costs into account creates a strong pressure for more types of RGCs with thinner axons and lower spike rates, consistent with a parallel processing model.

## Parallel Processing Models

L vs. M midget RGCs receptive fields are near optimal for high acuity spatial vision and are poorly suited for encoding hue. These facts plus the computational complexity required to separate hue from spatial information from L vs. M midget RGCs and a newer understanding of information processing in the retina has led to the suggestion of an alternative hypothesis: that the L vs. M midget RGCs’ only serve spatial vision – the function for which they are optimized – and they do not contribute to red-green hue perception. According to this idea, the front-end computations for hue perception are served, in parallel, by a second population of RGCs that have receptive field properties that are specifically optimized as hue detectors (Rodieck, 1991; Calkins and Sterling, 1999; Schmidt et al., 2014; Neitz and Neitz, 2016). The “pixel density” of the L vs. M midget RGCs is high to serve high spatial acuity but, as introduced above, the proposed parallel set of hue detectors need to be only relatively sparse to recover surface reflectance with much lower spatial acuity.

### Separate Subtypes of Midget RGCs for Hue and Spatial Vision

If L vs. M midget RGCs mediate spatial vision, which RGCs encode color? To match the acuity of our hue perception, an undiscovered RGC type would need roughly the sampling density of the S-cone mosaic (Mullen, 1985; Calkins and Sterling, 1999). The lack of alternative hue encoders makes midget RGCs an obvious candidate. We have proposed that the four fundamental hues are encoded by a small subset of L vs. M midget RGCs receiving input from neighboring S-cones (Figure 2F; Schmidt et al., 2014). The resulting L + S vs. M and M + S vs. L midget RGCs match the cone inputs for the four fundamental hues, as well as a population of rare RGCs (De Monasterio and Gouras, 1975; De Monasterio et al., 1975) and LGN neurons (Derrington et al., 1984; Tailby et al., 2008). These rare RGCs should not be ignored, as a potential hue-encoding RGC type needs to be only ∼5–10% of foveal RGCs to match color acuity (Calkins and Sterling, 1999).

Each S-cone has a surround created by S-cone-preferring HII horizontal cells. Hue-encoding receptive fields are proposed to arise from the superposition of the S-cone center-surround receptive field with the L vs. M cone center-surround. These two are predicted to be combined by feedforward synapses (Puller et al., 2014) from HII horizontal cells to L vs. M midget bipolar cells. The result simultaneously creates the S-cone input to L vs. M opponent cells and double opponency required to create nearly ideal hue-encoding RGCs (discussed in detail in Neitz and Neitz, 2016). Indeed, computational models of such color-coding midget RGCs can account for previously unexplained color phenomena, such as unique hues and variations in hue perception with L/M-cone ratios (Schmidt et al., 2016).

Key strengths of this parallel processing hypothesis are its simplicity and specificity. All the key features of ideal hue-encoding neurons are proposed to be created in the retina simply by feed-forward from HII horizontal cells at the level of the bipolar cells in a single step as opposed to the idea of multiple stages at unspecified higher levels. The predicted mechanism for a parallel set of double opponent neurons includes specific cell types, neurotransmitters, and biophysical mechanisms (Puller et al., 2014). While this level of detail may invite additional criticism, it also generates testable predictions that can be addressed by experiment. In contrast, the DeValois and DeValois model specified the computations for their “de-multiplexing” neurons, but not the underlying neural substrates.

### Recent Research Supporting Parallel Processing Models

The parallel processing approach draws from the idea that each RGC’s receptive field acts as a feature detector, tuned to extract a specific type of visual information, such as direction, defocus, edges or hue. From this perspective, L vs. M midget RGCs that respond equally to red–green and black–white edges are not multiplexing, nor even confounding, red–green and black–white signals. Rather, they are reliably signaling a particular feature – the presence or absence of an edge. Accordingly, hue-encoding RGCs are signaling a different feature – the detection of a specific spectral reflectance distribution (Figure 3A). Importantly, these RGCs would not be directly responsible for percepts of hue and edges, but instead we are proposing that they serve as front-end mechanisms for making these computations.

A particularly influential line of evidence has been provided by high-precision psychophysics experiments enabled by the development of adaptive optics systems capable of delivering small spots of light while simultaneously imaging the underlying mosaic of cones (Harmening et al., 2014). Early experiments investigating spatial acuity found individual midget RGCs set the limit for spatial resolution (Rossi and Roorda, 2010). These results are inconsistent with models proposing midget RGC outputs are combined to “de-multiplex” color and spatial information. The loss of spatial information from the convergence in Figure 2D can never be recovered at higher levels in the visual pathway.

The unprecedented precision provided by adaptive optics imaging systems combined with recent advances in eye tracking and cone type classification (Sabesan et al., 2015) have enabled highly precise psychophysics experiments investigating the percepts resulting from single cones (reviewed by Kling et al., 2019). The responses are highly consistent and reflect activity in the midget RGCs with single cone centers (Schmidt et al., 2019). Consistent with parallel processing of hue and spatial information by separate types of midget RGCs, stimulation of most L/M-cones in the central retina results in percepts of white, with only a small subset eliciting color percepts (Figure 3B; Sabesan et al., 2016; Schmidt et al., 2018a, b). Further, the homogeneity of the surrounding cone type had no effect on which cones were associated with a perceived color, arguing against the idea that midget RGCs with strong L vs. M opponency serve hue perception. These experiments were the first to target stimuli to single cones of a known type and represent a major advance in linking perception to underlying neural substrates in awake, behaving humans and the results will undoubtedly continue to challenge long-held assumptions.

## How Does the Cortex Use Wavelength Information?

Hue perception is just one of many functions that uses wavelength information. For example, the retina contains photopigments such as melanopsin and neuropsin, which carry additional wavelength information, but have no impact on the dimensionality of color vision (Horiguchi et al., 2013; Buhr et al., 2015). There are many examples of neurons carrying temporal, spatial or spectral information that is not extracted for visual perception, including color-opponent V1 neurons responding to chromatic stimuli that are not perceived (Gur and Snodderly, 1997; Jiang et al., 2007).

In fact, many RGCs do not contribute to conscious perception at all, but instead mediate functions such as visually guided movements or circadian photoentrainment (for review, see Neitz and Neitz, 2016). Wavelength information is extracted by several types of spectrally opponent RGCs for many functions other than color vision. For example, circadian rhythm photoentrainment and the pupillary light reflex are mediated by intrinsically photosensitive RGCs (reviewed in Do and Yau, 2010). Their receptive fields match the wavelength-encoding, single opponent receptive fields discussed above (Figure 2A; Dacey et al., 2005) – ideal for measuring the changes in chromaticity of ambient light throughout the day (Pauers et al., 2012; Spitschan et al., 2017) but they do not contribute to hue perception.

Several lines of evidence indicate that the ability to detect red-green edges is a distinct feature encoded separately from the ability to classify the appearance of lights as red or green. For example, patients with cerebral achromatopsia who suffer a total loss of hue perception, but still can detect chromatic borders, perceive shape from color and discriminate the direction in which colored patterns move (Cowey and Heywood, 1997). The existence of multiple mechanisms and uses for wavelength information also seems evident when comparing the cone inputs mediating color detection and color appearance. The studies identifying L + S vs. M and M + S vs. L as the cone inputs to hue perception measured color appearance (Wooten and Werner, 1979; Drum, 1989; Webster et al., 2000a; Schmidt et al., 2016). However, the classic psychophysical experiments that identified L vs. M and S vs. L + M as the “cardinal directions of color space” (Krauskopf et al., 1982), measured detection. Krauskopf et al. (1982) noted the disparity between their cardinal directions and the red-green (L + S vs. M) and blue-yellow (M + S vs. L) hue axes of color appearance and later questioned the evidence for cardinal mechanisms (Krauskopf, 1997).

There is common ground between multiplexing and parallel processing models. In discussing the abundance of chromatic cortical neurons, DeValois and DeValois argue that only a few are responsible for the specification of color, while the majority instead use color information to specify the spatial (or other) characteristics of stimuli. A problem was a lack of agreement on which cells were relevant for hue perception. Though their proposed color transformations were not consistent with the majority of published cortical color tuning studies, DeValois and DeValois pointed out inconsistencies in the literature and claimed one could “cite some cortical study in support of (or against) almost any suggestion about cortical color processing” (De Valois and De Valois, 1993) We argue a similar situation exists today in the retina where different studies can be sited in support or against the existence of S-cone inputs to midget RGCs [for example, compare the cone opponency reported by De Monasterio and Gouras (1975), Sun et al. (2006), and Field et al. (2010)].

## Future Directions

Both the parallel processing and multiplexing models would benefit from experiments linking the theories to their underlying neural substrates. However, an overarching difficulty for resolving the controversy over parallel vs. multiplexing theories is that each point of view reflects a deep-seated theoretical conviction. For those preferring the multiplexing view of L vs. M midget RGCs, “If the color signal is extractable, it makes little sense not to use it” (Billock et al., 1996). From a parallel processing standpoint, encoding color and spatial vision, two of the most fundamental aspects of visual perception, in a single binary channel makes little sense (Calkins and Sterling, 1999) and the information gained must outweigh the cost of extracting a color signal (Laughlin et al., 1998).

Thus, further experiments to characterize the response properties of visual neurons alone are not going to settle the controversy. Initial surveys of cone inputs to neurons in the retinal and LGN reported S-cone input to a subset of L vs. M neurons (De Monasterio and Gouras, 1975; De Monasterio et al., 1975; Derrington et al., 1984) and later surveys confirmed these findings (Tailby et al., 2008; Field et al., 2010). However, skeptics of the parallel processing models favor a study by Sun et al. (2006) in which the authors recorded from a large population of midget RGCs and concluded S-cone input was unlikely (Sun et al., 2006). An underlying problem is that the answers depend on how you ask the question. Results from receptive field measurements are a function of stimulus choice. For example, a full-field stimulus (Lee et al., 1998) may have reduced S-cone responses by driving the antagonistic S-cone surround receptive field mediated by HII horizontal cell feedback (Dacey et al., 1996). Indeed, the Sun et al. (2006) experiments did not detect S-OFF midget RGCs, despite a growing consensus that these neurons make up 5–10% of OFF midget RGCs in the macaque central retina (Klug et al., 2003; Field et al., 2010; Tsukamoto and Omi, 2015; Patterson et al., 2019). Taken together, these results further demonstrate the need to account for both the spatial and spectral dimensions of midget RGC receptive fields.

Consideration of underlying theoretical perspectives and stimulus biases will be essential for designing future experiments linking color vision models to their underlying neural substrates. Also, a broader perspective may help answer the larger questions about how our eye and brain process visual information. Hopefully, future research using cutting-edge technologies will provide satisfying explanations for long unanswered mysteries of vision.

## Author Contributions

SP wrote the manuscript. MN and JN edited the final version of the manuscript.

## Conflict of Interest Statement

The authors declare that the research was conducted in the absence of any commercial or financial relationships that could be construed as a potential conflict of interest.
